# *Cymbopogon* Species; Ethnopharmacology, Phytochemistry and the Pharmacological Importance

**DOI:** 10.3390/molecules20057438

**Published:** 2015-04-23

**Authors:** Opeyemi Avoseh, Opeoluwa Oyedeji, Pamela Rungqu, Benedicta Nkeh-Chungag, Adebola Oyedeji

**Affiliations:** 1 Chemistry Department, University of Fort Hare, 5700 Alice, South Africa; E-Mails: seavoseh@gmail.com (O.A.); 200800815@ufh.ac.za (P.R.); 2 Department of Zoology, Walter Sisulu University, 5099 Mthatha, South Africa; E-Mail: bnkehchungag@wsu.ac.za; 3 Department of Chemistry, Walter Sisulu University, 5099 Mthatha, South Africa; E-Mail: aoyedeji@wsu.ac.za

**Keywords:** *Cymbopogon*, ethnopharmacology, secondary metabolites, terpenes, chemo-types

## Abstract

*Cymbopogon* genus is a member of the family of Gramineae which are herbs known worldwide for their high essential oil content. They are widely distributed across all continents where they are used for various purposes. The commercial and medicinal uses of the various species of *Cymbopogon* are well documented. Ethnopharmacology evidence shows that they possess a wide array of properties that justifies their use for pest control, in cosmetics and as anti-inflammation agents. These plants may also hold promise as potent anti-tumor and chemopreventive drugs. The chemo-types from this genus have been used as biomarkers for their identification and classification. Pharmacological applications of *Cymbopogon citratus* are well exploited, though studies show that other species may also useful pharmaceutically. Hence this literature review intends to discuss these species and explore their potential economic importance.

## 1. Introduction

The presence of secondary metabolites in plants is characterized by their ability to provide defenses against biotic and abiotic stress [1]. The mechanism of defense varies from plant to plant, their environmental conditions and climatic variations. However, the presence of these metabolites in plant are usually in minimum amounts though several molecular techniques are available to either increase or decrease the quantity of a particular metabolite by blocking competitive pathways and enriching metabolites of choice [2]. Terpenes, alkaloids (*N*-containing compounds) and phenolics constitute the largest groups of secondary metabolites. The shikimic acid pathway is the basis of the biosynthesis of phenolics while the terpenes which are comprised of isoprene units arise from the mevalonate pathway [3]. Aspirin (**1**) from white willow, quinine (**2**) from the cinchona plant and artemisinin (**3**) from *Artemisia annum* are all plant secondary metabolites. The biological application of these metabolites as therapeutic agents for a broad spectrum of ailments and the microbial infections has been salutary in human history.

The genus *Cymbopogon* is widely distributed in the tropical and subtropical regions of Africa, Asia and America. Comprised of 144 species, this genus is famous for its high content of essential oils which have been used for cosmetics, pharmaceuticals, and perfumery applications [4]. Two main species, *C. flexuosus* and *C. citratus* (lemongrass) are commercially cultivated in the Democratic Republic of Congo (DRC), Madagascar, and the Comoros Island. However, the leading exporter of these plants is Guatemala, trading about 250,000 kg per year and while the USSR sells about 70,000 kg per year [5].

The commercial value of some *Cymbopogon* species is further enhanced by their ability to grow in moderate and extremely harsh climatic conditions [6]. In environments where they are not used for cosmetics, drug or perfumery, such as in the Eastern Cape Province of South Africa, these plants have found a good application as roof thatches and grass brooms [7].

## 2. Ethnopharmacology of *Cymbopogon* Species

Traditional applications of *Cymbopogon* genus in different countries shows high applicability as a common tea, medicinal supplement, insect repellant, insecticide, in flu control, and as anti-inflammatory and analgesic. Table 1 shows the common names of some species, their relevance and how they are applied. *C. citratus* is ranked as one of the most widely distributed of the genus which is used in every part of the world. Its applications in Nigeria include cures for upset stomach, malaria therapy, insect repellent and as an antioxidant (tea) [8]. *C. citratus* and *C. flexuosus* are the prevailing species in Eastern and Western India and have been used locally in cosmetics, insecticides, and for the treatment of digestive disorders and fevers [9,10].

**Table 1:** Several *Cymbopogon* species, common name, regions, plant part used and the uses.

*Species*	Region	Common Name	Parts	Medicinal Uses	References
***C. nardus (L.)* Rendle**	India	Citronella oil	Leaves	Insect repellent and as perfumes	[11]
***C. parkeri* Stapf**	Pakistan	Lemon grass	Aerial	Antiseptic and stomachic treatment	[12]
***C. excavatus* Hoscht**	South Africa	Bread-leavened Turpentine grass	Sheaths	Used as insecticides	[13]
***C. olivieri(*Boss*)***	Pakistan	Pputar	Aerial	Pyretic, vomit, diuretic, rheumatism, and as anti-malaria condiment.	[14,15]
***C. validus(*Stapf*)***	Eastern and Southern Africa	African bluegrass	Essential oils	skin toner, anti-ageing in men, fumigant and for rodent control	[16]
***C. winterianus(*Jowitt*)***	Brazil	Java grass	Fleshy leaves	Treatment of epilepsy and anxiety	[17]
***C. marginatus(*Steud.)**	South Africa	Lemon-Scented grass	Root	They are used as moth repellent	[18]
***C. citratus* Stapf**	India	Lemon grass	Aerial	Fever, digestive disorders	[9]
	Nigeria	Lemon grass	Leaves	Diabetes, inflammation and nerve disorders	[8]
	Argentina	Limonaria	Leaves	Against cold and flu, and digestive complaints, stomach upsets and as decoction with other plants for malaria	[19]
	Cuba	Cana Santa	Leaves		[20]
	Costa Rica	Grass tea	Leaves	To relieve cough, carminative, expectorant and depurative	[21]
	Colombia	Limonaria	Rhizome	It is chewed and used as toothbrush and for pest control.	[22,23]
	Brazil	Capimsanto	Leaves	Anxiolytic and anti-hypertensive	[24]
	Trinidad & Tobago	“fever grass”	Grass and rhizomes	The teas from it are used to treat cold, flu, fever and diabetes	[24]
***C. giganteus(*Hochst.) Chiov.**	Cameroon	Tsauri grass	decoctions of leaves and flowers	Cough and arterial hypertension	[25]
***C. ambiguous* (Hack.) A. Camus.**	Australia	Native Lemon Grass	Leaves and stems	Headache remedy, chest infections, muscle cramp and Scabies	[26,27]
***C. procerus* (R.Br.) Domin**	Australia	Scent grass	Leaves and stems	Leaves and stem are pounded and used as medicinal body wash used for headache	[28]
***C. flexuosus* (Nees ex Steud.) Wats.**	India	Lemon grass	Leaves	Cosmetics, antiseptic and for treatment of fever	[10]
***C. pendulus* (Nees ex Steud.) Wats.**	India	Jammu Lemongrass	Leaves	Antiseptic and for perfumery	[29]
***C. scheonanthus* (L.) Spreng**	Saudi Arabia	Ethkher	Leaves	Antidiarrheal, to treat fever, treatment of jaundice and tonic	[30]
***C. obtectus* (S.T. Blake)**	Central Australia	Silky-heads	Mixture	Cold and flu, headaches, fever and sore throat	[27]
***C. proximus* (Stapf.)**	Egypt	Halfabar	Leaves	Expulsion of renal and ureteric calculi	[31]
***Cymbopogon refractus* (R.Brown) A. Camus.**	Australia	Barbed wire grass	Leaves	Feed for animals	[32]
***C. densiflorus* (Steud.) Stapf**	Congo	Lemongrass	Leaves and rhizome	Employed against asthma, epilepsy, abdominal cramps and pains and also for interpreting dreams by witch doctors.	[33,34]
***C. jwarancusa(*Jones) Schult.**	Egypt	Thé Limon	The whole plant	Condiment and for medicinal purpose	[35]

In the Middle East, *C. olivierri* and *C. parkeri* are more predominant, and they are used as antiseptics, anti-malarial condiments, diuretics and also to cure rheumatism [12,14,15]. The high amounts of volatile compounds from these species are responsible for their diverse uses.

**Figure 1:**
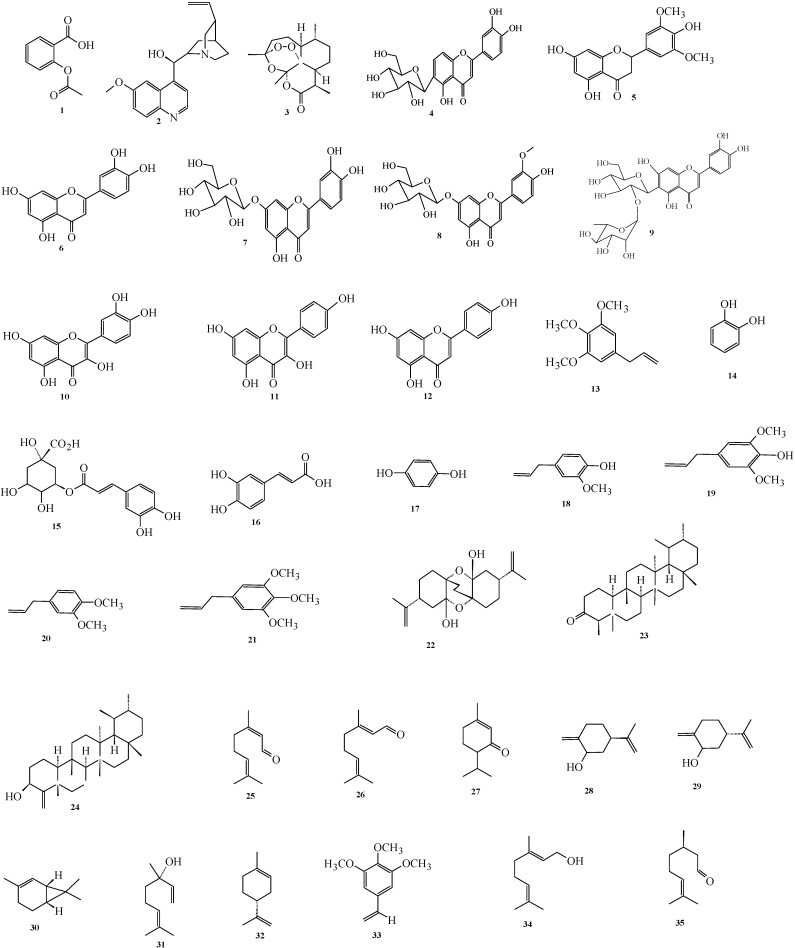
Flavonoids and triterpenoids from *Cymbopogon* species.

## 3. Phytochemistry

The enormous information gathered from the ethno-pharmacological applications of *Cymbopogons* begged the investigation of its chemical constituents. These studies have led to the isolation of alkaloids, volatile and non-volatile terpenoids, flavonoids, carotenoids and tannins from every part of these plants. Figure 1 displays some of the compounds isolated from *Cymbopogon* species.

### 3.1. Alkaloids

The rhizome of *C. citratus* from Nigeria was reported to contain about 0.52% alkaloids from 300 g plant material [36].

### 3.2. Flavonoids

This class of compounds has potent antioxidant properties. Some of the flavonoids isolated from *Cymbopogon* species are presented in Figure 1. Isoorientin (**4**) and tricin (**5**) were isolated from the dichloromethane extract of *C. parkeri* [37], evaluation of these two compounds revealed their muscle relaxation activity [38]. Isolation of luteolin (**6**), luteolin 7-*O*-glucoside (cynaroside) (**7**), isoscoparin (**8**) and 2''-*O*-rhamnosyl isoorientin (**9**) from the leaves and rhizomes of *C. citratus* has been reported. Other flavonoid compounds isolated from the aerial parts of *C. citratus* are quercetin (**10**), kaempferol (**11**) and apigenin (**12**) [39], isolated elimicin (**13**), catechol (**14**), chlorogenic acid (**15**), caffeic acid (**16**) and hydroquinone (**17**) from the aerial parts of the same species. Isolation of 4-phenylpropanoids from Australian species of *C. ambiguus* has been reported. These compounds are eugenol (4-allyl-2-methoxyphenol) (**18**); elemicin (5-allyl-1,2,3-trimethoxybenzene) (**19**); eugenol methylether (4-allyl-1,2-dimethoxybenzene) (**20**) and *trans-*iso-elemicin (1,2,3-trimethoxy-5-(1-propenyl) benzene) (**21**) and all these isolates exhibited good inhibition activity against ADP-induced human platelet serotonin release which is associated with headaches [26].

### 3.3. Cymbopogon Terpenoids 

#### 3.3.1. Non-Volatile Terpenoids

Plants in the *Cymbopogon* genus contain large amounts of volatile terpenoids though a few species from this genus are reported to contain non-volatile terpenoids as well. Bottini *et al*. [40] isolated a novel bis-monoterpenoid named cymbodiacetal (**22**) from *C. martinii*. The triterpenoids cymbopogone (**23**) and cymbopogonol (**24**) (Figure 1) were also reported from the leaves of *C. citratus* [41].

#### 3.3.2. Volatile Terpenoids of *Cymbopogon* Species

Different chemotypes of *Cymbopogon* species contain varying major compounds such as citral, geraniol, citronellol, piperitone and elemin (Table 2). In the literature, the majority of the *C. citratus* analysed showed a remarkably high percentage of neral (**25**) and geranial (**26**). Analysis of *C. citratus* species from Brazil [42], India [43], West and Eastern Africa [43,44,45,46,47,48,49] and Asia [50] showed the high value of neral and geranial chemotypes. A special distinguishing feature between *C. citratus* of African origin is the high amount of myrcene observed in them [44,45,46,47,48,49]. High occurance of piperitone (**27**) characterizes the oils of *C. parkeri* and *C. olivieri* from Iran. Jiroveltz *et al.* [25] reported a significant presence of *cis-p*-mentha-1(7),8-dien-2-ol (**28**) and its isomer *trans*-*p*-mentha-1(7),8-dien-2-ol (**29**) in the oils of *C. giganteus* from Cameroon [25]. Predominant components observed in other *Cymbopogon* species essential oils from around the world include δ-2-carene (**30**) in *C. proximus* from Cameroon [51], linalool (**31**) from Malaysia’s *C. nardus* [52], limonene (**32**) in *C. schoenanthus* (Tunisia) and *C*. *giganteus* (Burkina Faso) [46] and elemicin (**33**) from the oils of *C. pendulus* from India [53]. Observation of the oil of *C. winterianus* from different parts of Brazil showed two major chemotypes based on the amount of geraniol (**34**) and citronellal (**35**) [17,54,55,56]. 

**Table 2:** Major components observed in some *Cymbopogon* species.

Compound	*Species*	Country/Region	Major %	References
cis-*p*-mentha-1(7),8-dien-2-ol (C_10_H_16_O)	*C. giganteus(F)*	Cameroon	22.8	[25]
		Burkina Faso	12.0	[46]
		Madagascar	19.0	[57]
trans-*p*-mentha-1(7),8-dien-2-ol	*C. giganteus*	Cameroon	26.5	[25]
	*C. giganteus*	Burkina Faso	14.2	[46]
	*C. densiflorus*	Zambia	11.1	[57]
	*C. giganteus*	Madagascar	22.4	[56]
Limonene (C_10_H_16_)	*C. giganteus*	Cameroon	7.4	[25]
	*C.giganteus*	Burkina Faso	42.0	[46]
	*C. proximus*	Burkina Faso	3.9	[51]
	*C. schoenanthus*	Tunisia	24.2	[58]
Elemicin (C_12_H_16_O_3_)	*C. pendulus*	India	53.7	[53]
α-Pinene (C_10_H_16_)	*C. pendulus*	India	6.1	[53]
Camphene (C_10_H_16_)	*C. pendulus*	India	9.1	[53]
	*C.winterianus*	India	8.0	[59]
Geranial (C_10_H_16_O)	*C. flexuosus*	India (Kumauon region)	33.1	[60]
		India (Bilhar)	42.4	[43]
	*C. citratus*	Burkina Faso	48.1	[46]
		Brazil	50.0	[42]
		Egypt	40.72	[61]
		Zambia	39.0	[47]
		Kenya	39.53	[57]
		Benin republic	27.04	[62]
		Nigeria	33.7	[44]
		Angola	40.55	[63]
		Congo Brazaville	48.88	[45]
		Ivory Coast	34.0	[45]
		Mali	45.3	[45]
		Iran	39.16	[50]
	*C. winterianus*	S.E. Brazil	8.05	[55]
Neral (C_10_H_16_O)	*C. flexuosus*	India	30.0	[60]
		Burkina Faso	34.6	[46]
		India (Bilhar)	29.8	[43]
		Brazil (North)	30.1	[42]
		Egypt	34.98	[61]
		Zambia	29.4	[47]
		Kenya	33.31	[48]
	*C. giganteus*	Benin republic	19.93	[62]
		Nigeria	26.5	[44]
	*C. citratus*	Angola	28.26
		Malaysia	50.81	[64]
		Congo Brazzaville	36.24	[49]
		Brazil	4.53	[17]
		Ivory Coast	32.5	[45]
		Mali	26.3	[45]
		Iran	30.95	[50]
Geranyl acetate (C_12_H_20_O_2_)	*C. flexuosus*	India	12.0	[60]
Linalool (C_10_H_18_O)	*C. flexuosus*	India	2.6	[60]
	*C.winterianus*	India	1.5	[59]
	*C. martini*	India	2.0	[65]
	*C. nardus*	Malaysia	11.0	[52]
Geraniol (C_10_H_18_O)	*C. winterianus*	India	23.9	[59]
	*C. martinii*	India	84.16	[65]
	*C. winterianus*	Brazil	32.82	[17]
		Brazil (para state)	16.2	[54]
	*C. winterianus*	S.E Brazil	40.06	[55]
Citronellal (C_10_H_18_O)	*C.winterianus*	India	32.7	[59]
	*C. nardus*	Malaysia	29.6	[52]
	*C. winterianus*	Brazil	36.19	[17]
	*C. winterianus*	Brazil (para state)	26.5	[54]
	*C. winterianus*	S.E. Brazil	27.44	[55]
Citronellol (C_10_H_20_O)	*C. winterianus*	India	15.9	[59]
	*C. winterianus*	Brazil	11.34	[17]
	*C. winterianus*	Brazil (Para state)	7.3	[54]
	*C. winterianus*	S.E. Brazil	10.45	[55]
Myrcene (C_10_H_16_)	*C. citratus C. citratus C. citratus*	Burkina Faso	11.0	[46]
		Egypt	15.69	[61]
		Zambia	18.0	[47]
		Benin republic	27.83	[62]
		Nigeria	25.3	[44]
		Angola	10.57	[63]
		Ivory Coast	18.1	[45]
		Mali	9.1	[45]
Selina-6-en-4-ol (C_15_H_26_O)	*C. citratus*	Brazil	27.8	[42]
α-Cadinol (C_15_H_26_O)	*C. citratus*	Brazil	8.2	[42]
Piperitone (C_10_H_16_O)	*C. olivieri*	Iran	72.8	[14]
	*C. parkeri*	Iran	80.8	[12]
	*C. proximus*	Burkina Faso	59.1	[51]
4-Carene (C_10_H_16_)	*C. olivieri*	Iran	11.8	[12]
Germacrene-D (C_15_H_24_)	*C. parkeri*	Iran	5.1	[11]
δ-2-Carene (C_10_H_16_)	*C. proximus*	Burkina Faso	22.3	[51]
β-Phellandrene (C_10_H_16_)	*C. schoenanthus*	Tunisia	13.4	[58]

#### 3.4. Tannins

A literature search on the phytochemical screening of *C. citratus* also reveals the presence of tannins, however, very little effort has been made in the isolation of these compounds despite the appreciable amounts reported through quantitative phytochemical tests. Figueirinha *et al.* fractionated extracts of the species collected from Portugal and reported about 10 mg dry weight of hydrolysable tannins (prothocyanidins) [66] while *C. citratus* from Nigeria showed about 0.6% of tannins [36]. *C. citratus* is the single species of *Cymbopogon* which is most exploited for its tannin content.

## 4. Pharmacology

Several bioassays have confirmed the potency of *Cymbopogon* species for their several uses (Table 3). *C. citratus* was found to have chemoprotective activity by preventing of diethylnitrosamine (DEN)-initiated hepatocellular lesions in rats [67]. In South Africa, extract from *C.* c*itratus* was applied for treatment of oral thrush in patients who tested positive to HIV/AIDS and proved effective [68].

Insecticidal activity is one of the biological effects of most plant of the *Cymbopogon* genus; it is either applied as pest control for stored crops or as mosquito repellent/ insecticide. The essential oils of *C. martinii* have been studied and found to display high anthelmintic activity against *Caenorhabditis elegans* at ED_50_ value of 125.4 µg/mL, *C schoenanthus, C. giganteus and C. citratus* essential oils from Benin Republic in West Africa all displayed about 100% mortality rate against adult *Anopheles gambiae* [69]. The essential oil from *C. winterianus* caused a dose dependent mortality of *Culex quinquefasciatus* with LC_50_ of 0.9% [70].

The anticancer properties of *Cymbopogon* species have also been studied. The essential oils of *C. flexuosus* was effective in inhibiting the growth and killing of Ehrlich and Sarcoma-180 tumors cells. In this study, it was discovered that at a dose of 200 mg/kg, Ehrlich solid tumor inhibition was about 57.83% compared to the 45.23% inhibition observed with 5-fluorouracil (22 mg/kg) [71]. Inhibition of early phase of hepatocarcinogenesis was also observed in *C. citratus* [67]. Positive results in several other bioassays such as antiprotozoal, anti-inflammatory, antimicrobial, anti-bacterial, anti-diabetic, anticholinesterase, molluscidal, antifungal and larvicidal activity are also prominent with *Cymbopogon* species as outlined in Table 3.

**Table 3:** Pharmacological evidence of some *Cymbopogon* species.

Cymbopogon Species	Pharmacology	Activity	References
*C. citratus*	Cytotoxicity	Shows high toxicity against Chinese Hamster Ovary (CHO) cells (IC_50_ = 10.63 μg/mL) and moderately toxic against human fibroblast cell line 138 (W138) cells (IC_50_ = 39.77 μg/mL).	[72]
	Insecticidal	LC_50_ of 48.6 μL/L against housefly larvae	[43]
	Neurobehavioral effects	Ability to be active as sedative, anxiolytic and anticonvulsant agent	[73]
	Antitrypanosomal	Modest activity against *Trypanasoma brucei* IC_50_ = 1.837 ± 0.13 μg/mL	[72]
	Anti-diabetic	Shows activity against poloxamer-407 induced type 2 diabetic (T2D) in Wistar rats	[43]
	HIV/AIDS	As a highly effective control for oral thrush in HIV/AIDS victims in South Africa	[68]
	Larvicidal activity	It shows high inhibition and mortality rate against larva of *A. aegypti*	[74]
	Chemopreventive activity	Inhibits the early phase of hepatocarcinogenesis in rats	[67]
	Anti-inflammations	Hexane extract inhibited iNOS (inducible nitric oxide synthase)expression, NO (nitric oxide) production and various LPS (lipopolysaccharide)-induced pathways	[75]
*C. schoenanthus*	Antioxidant(DPPH)	36%–73.8% activity per 2 μL of oil	[58]
	Acetylcholinesterase inhibitory	IC_50_ = 0.26 ± 0.03 mg mL^−1^	[58]
	Insecticidal activity	2.7 μL/L obtained for LC_50_ against *Callosobruchus maculatus*	[76]
*C. winterianus*	Moluscidal	LC_90_ = 97.0mg/L and LC_50_ = 54.0 mg/L	[54]
	Larvicidal	LC _50_ = 181.0mg/L	[54]
	Anti-fungal	Inhibited the growth of 15 strains of *Candida albicans* at concentrations of 625 μg/mL and 1250 μg/mL	[77]
*C. giganteus*	Antimicrobial	High activity against gram +ve and gram −ve bacteria	[25]
	Cytotoxicity	Low cytotoxicity against CHO cells and the human non cancer fibroblast cell line (W138)	[72]
	Anti-trypanosomal	IC_50_ = 0.25 ± 0.11 μg/mL against *Trypanasoma brucei*	[72]
	Antiplasmodial	High activity with an IC_50_ ≤ 20 μg/mL	[72]
*C. pendulus*	Antifungal	Strong activity against *Microsporum audouinii*, *Trichophyton rubrum* and *Epidermophyton floccosum* at 100% for all the species	[78]
*C. flexuosus*	Chemopreventive	Potent *in vivo* activity against Ehrlich and Sarcoma-180 tumors.	[71]
*C. densiflorus Stapf*	Antibacterial	Gram-negative bacteria. MICs were found to be between 250 and 500 ppm for the Gram-positive and between 500 and 1000 ppm for the Gram-negative bacteria	[79]
*C. ambiguus*	Inflammatory	Inhibition of ADP-induced human platelet serotonin release in the cell.	[26]
*C. nardus*	Antibacterial	MIC values ranged from 0.244 µg/mL to 0.977 µg/mL when tested against the bacterial isolates	[52]
*C. nervatus*	Molluscidal activity	It inhibits *Biomphalaria pfeifferi* at LD_50_ of 213.099 ppm dose dependent	[80]
*C. olivieri*	Antimicrobial activity	Exhibited excellent antimicrobial activity against gram ±ve organisms	[14]

## 5. Conclusions

*Cymbopogon* species have been used as traditional medicine in many countries. Of all the species reviewed, *C. citratus* and *C. flexuosus* are the most widely used in traditional and in conventional medicine due to the pharmacological potential of their phytochemicals. The majority of these species contain a voluminous amount of essential oils which have shown several biological activities such as insecticidal, anti-protozoan, anticancer, anti-HIV, anti-inflammatory and anti-diabetes effects.
